# Flexible Polymer-Based Electronics for Human Health Monitoring: A Safety-Level-Oriented Review of Materials and Applications

**DOI:** 10.1007/s40820-025-02059-7

**Published:** 2026-01-21

**Authors:** Dan Xu, Yi Yang, Keiji Numata, Bo Pang

**Affiliations:** 1 Department of Material Chemistry, Graduate School of Engineering, Kyoto University, Kyoto, 615-8510 Japan; 2 Department of Food Science & Technology, National University of Singapore, Singapore, 117542 Singapore; 3 Biomacromolecules Research Team, RIKEN Center for Sustainable Resource Science, Wako, 351-0198 Japan

**Keywords:** Flexible devices, Polymers, Health monitoring, Safety-level-oriented, Interfacial designs

## Abstract

Health monitoring is becoming increasingly critical for disease prevention, early diagnosis, and high-quality living. Polymeric materials, with their mechanical flexibility, biocompatibility, and tunable biochemical properties, offer unique advantages for creating next-generation personalized devices. In recent years, flexible polymer-based platforms have shown remarkable potential to capture diverse physiological signals in both daily and clinical contexts, including electrophysiological, biochemical, mechanical, and thermal indicators. In this review, we introduce a safety-level-oriented framework to evaluate material and device strategies for health monitoring, spanning the continuum from noninvasive wearables to deeply embedded implants. Physiological signals are systematically classified by use case, and application-specific requirements such as stability, comfort, and long-term compatibility are highlighted as critical factors guiding the selection of polymers, interfacial designs, and device architectures. Special emphasis is placed on mapping material types—including hydrogels, elastomers, and conductive composites—to their most suitable applications. Finally, we propose design principles for developing safe, functional, and adaptive polymer-based systems, aiming at reliable integration with the human body and enabling personalized, preventive healthcare.

## Highlights

- A safety-level-oriented framework is proposed to systematically classify polymer-based flexible health-monitoring devices from noninvasive to long-term implantable modalities.
- Material–safety relationships are elucidated by mapping hydrogels, elastomers, and conductive composites to modality-specific requirements in mechanical compliance, biochemical stability, electrical safety, and long-term biointegration.
- Time-scale-dependent design principles are summarized to guide future development of safe, adaptive, and clinically translatable polymer-based monitoring systems.

## Graphical abstract

**Figure:**
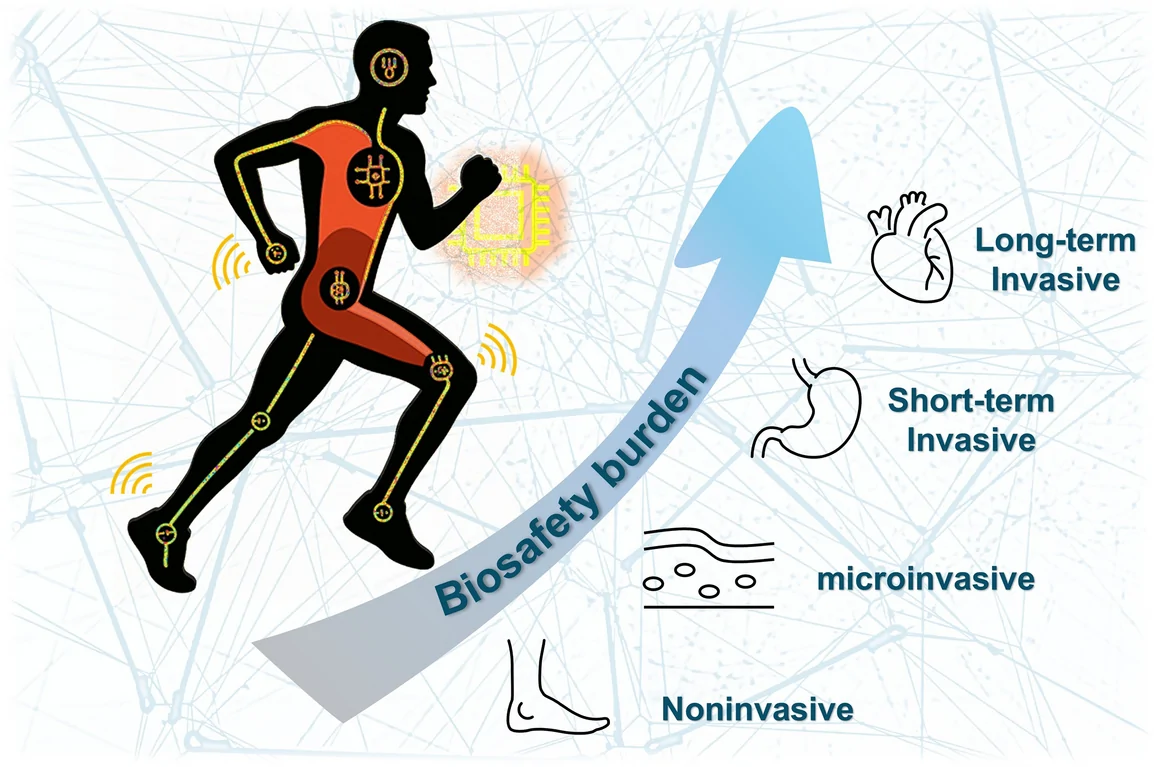

## Introduction

Health monitoring plays an increasingly vital role in modern disease prevention and the pursuit of high-quality living [1–4]. The rising prevalence of chronic disease, coupled with global population aging, has created an urgent demand for continuous, personalized, and preventive medical strategies [5–7]. In parallel, the rapid expansion of wearable technologies and digital healthcare platforms underscores the expectation that health‑monitoring devices operate reliably in both clinical and everyday environments [8]. Beyond episodic diagnostics, there is a shift toward longitudinal tracking of digital biomarkers—capturing physiology during sleep, exercise, and daily routines—to enable earlier intervention and more equitable access to care [9]. These societal and medical drivers set the stage for materials and device concepts that can bridge the gap between technological performance and human adaptability [10].

Against this backdrop, polymeric materials have emerged as particularly promising candidates for health‑monitoring devices [11, 12]. Their inherent mechanical flexibility, low modulus, and tunable biochemical functionality enable platforms that integrate more naturally with the human body [13]. Over the past few years, flexible polymer‑based devices have demonstrated the ability to capture electrophysiological [14] (e.g., electrocardiogram (ECG), electroencephalogram (EEG) and electromyogram (EMG), biomechanical (pressure, strain) [15], and biochemical (sweat or interstitial‑fluid analytes)) [16] signals across diverse scenarios. Unlike traditional rigid electronics, which often suffer from mechanical mismatch with soft tissues—leading to discomfort, unstable signal acquisition, or even tissue irritation—polymer‑based platforms provide intimate, conformable interfaces with skin, mucosa, and internal organs [17]. Hydrogels [18], elastomers [19], and conductive polymers [20], for instance, each contribute unique properties that collectively allow soft, biocompatible, and multifunctional integration. Furthermore, advances in stretchable interconnects, soft encapsulants, and textile‑grade substrates have improved device robustness and enabled unobtrusive form factors compatible with daily use [21, 22]. These advances highlight not only the technical feasibility of polymer‑based systems but also their relevance to unmet needs in real‑world healthcare.

However, the translation of such promising platforms requires considerations beyond conventional performance metrics. While sensitivity, selectivity, and durability remain crucial, safety has emerged as a decisive factor for real-world (i.e., clinically and daily-life relevant) deployment [23]. Biological safety spans both acute and chronic dimensions, including cytotoxicity, irritation and sensitization, fibrotic encapsulation, foreign-body responses, and long-term mechanical damage arising from modulus mismatch or interfacial friction [24, 25]. Chemical safety must be ensured by stable encapsulation, minimization of leachable species such as residual monomers, plasticizers, or degradation by-products, and careful control of electrolyte and solvent exposure [26–28]. Electrical safety requires robust insulation, strict adherence to leakage-current thresholds (as defined by IEC 60601‑1 standards), and fail-safe operation under physiologically relevant conditions such as perspiration, repetitive motion, and laundering [29, 30]. In addition, failure modes typical of soft materials—such as delamination at soft–hard interfaces [31], moisture-induced corrosion [32], ionic drift [33], and fatigue cracking under repeated strain [34]—directly impact safety, reliability, and data fidelity. Despite their importance, these concerns have often been treated as secondary to functional optimization in device development, creating a persistent gap between device capability and biological integration [35].

**Fig. 1:**
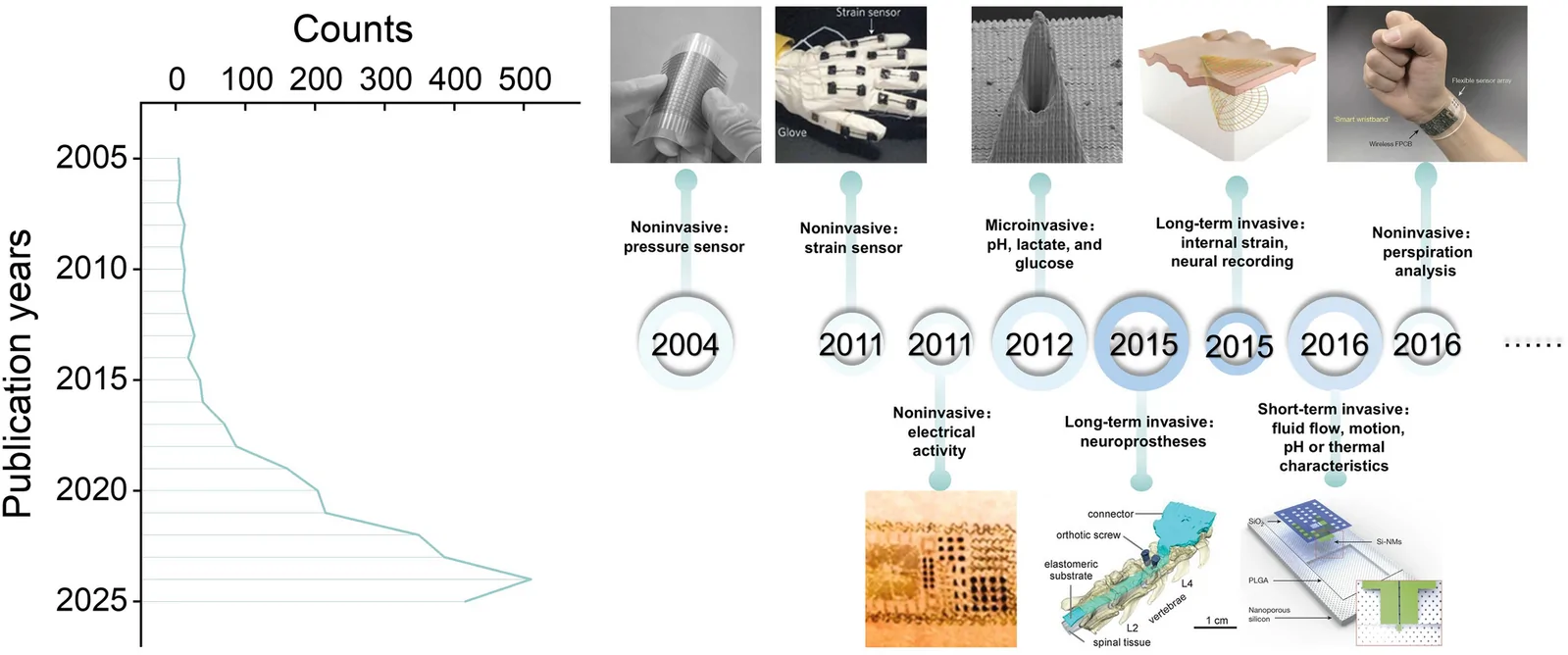
Rising publication trend and representative development timeline of flexible polymer-based electronics for human health monitoring. Publication counts were extracted from the Web of Science database, with the dataset updated to November 2025. Reproduced with permission. Copyright 2004, National Academy of Sciences [36]; Copyright 2011, Springer Nature [37]; Copyright 2011, The American Association for the Advancement of Science [38]; Copyright 2011 Elsevier [39]; Copyright 2015, The American Association for the Advancement of Science [40]; Copyright 2015, Springer Nature [41]; Copyright 2016, Springer Nature [42]; Copyright 2016, Springer Nature [43]

Importantly, these safety considerations have become increasingly prominent as the field has progressed from early noninvasive wearables to microinvasive platforms and, more recently, implantable bioelectronic systems. To contextualize this evolution, Fig. 1 summarizes both the rising publication trend and the key technological milestones that have shaped flexible polymer-based electronics for human health monitoring. These milestones reveal a steady shift toward deeper tissue interfaces, higher biosafety demands, and more sophisticated polymer architectures.

**Fig. 2:**
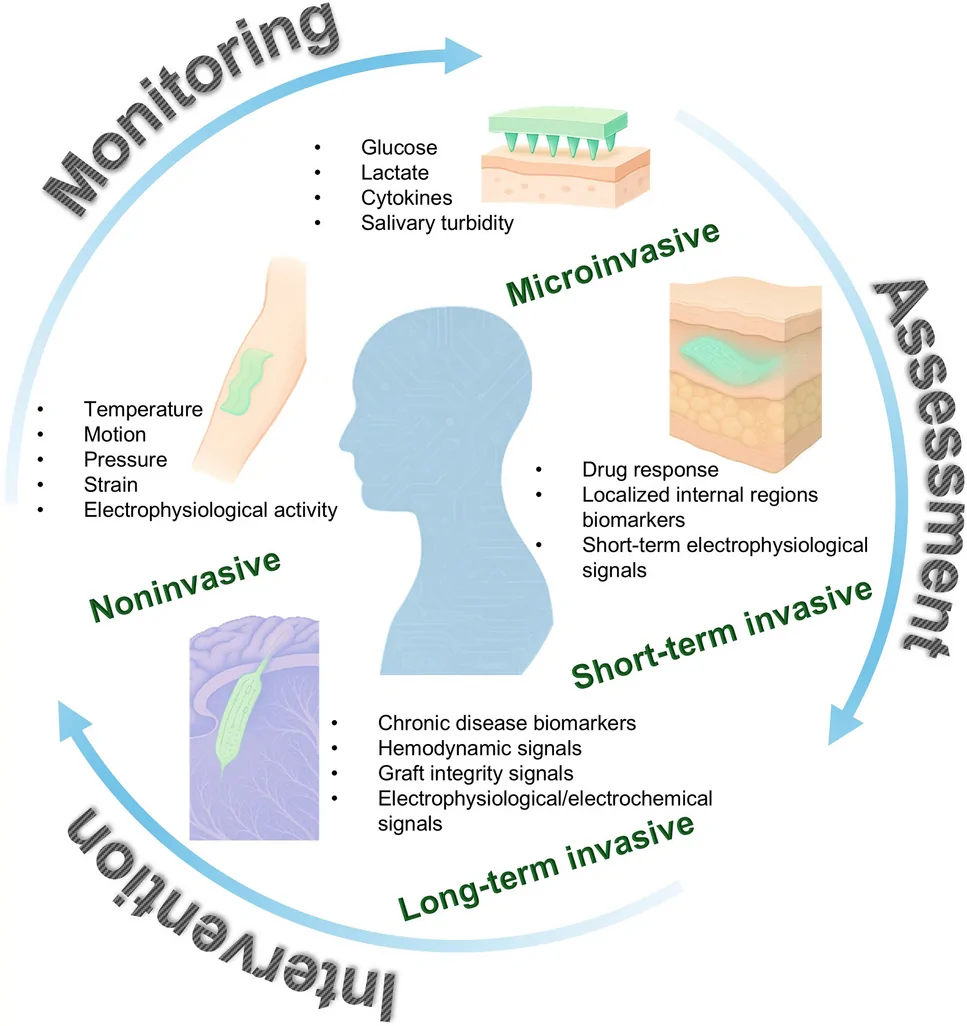
Illustration of safety-level-oriented electronics in human health monitoring

Building on this historical and technological context, this review introduces a safety-level-oriented framework for evaluating polymer-based flexible electronics for health monitoring. Devices are categorized according to their degree of invasiveness and duration of contact with the human body, ranging from noninvasive, microinvasive, and short-term implantable to long-term implantable systems, with corresponding biomarkers, within a closed-loop framework that involves monitoring, assessment, and intervention (Fig. 2). While previous reviews have catalogued flexible polymer-based wearables and implants primarily by material class or sensing function, none has provided an invasiveness- and contact-duration-based structure that links biosafety burden directly to polymer design. By contrast, our framework offers a modality-integrated, safety-driven perspective that complements existing reviews and uniquely captures the challenges faced by emerging soft, transient, and multifunctional devices. Each category imposes distinct functional and biosafety requirements—including mechanical compliance, chemical stability, degradation behavior, and immune tolerance—which directly dictate material selection and device architecture.

By aligning material design with safety-level requirements across diverse application scenarios, this review aims to bridge the gap between device performance and biological integration. Ultimately, the goal is to promote safer, smarter, and more adaptable health-monitoring systems capable of seamless operation in both clinical and everyday contexts.

## Safety-Level of Polymer Flexible Electronics in Human Health Monitoring

### Noninvasive Modality

**Fig. 3:**
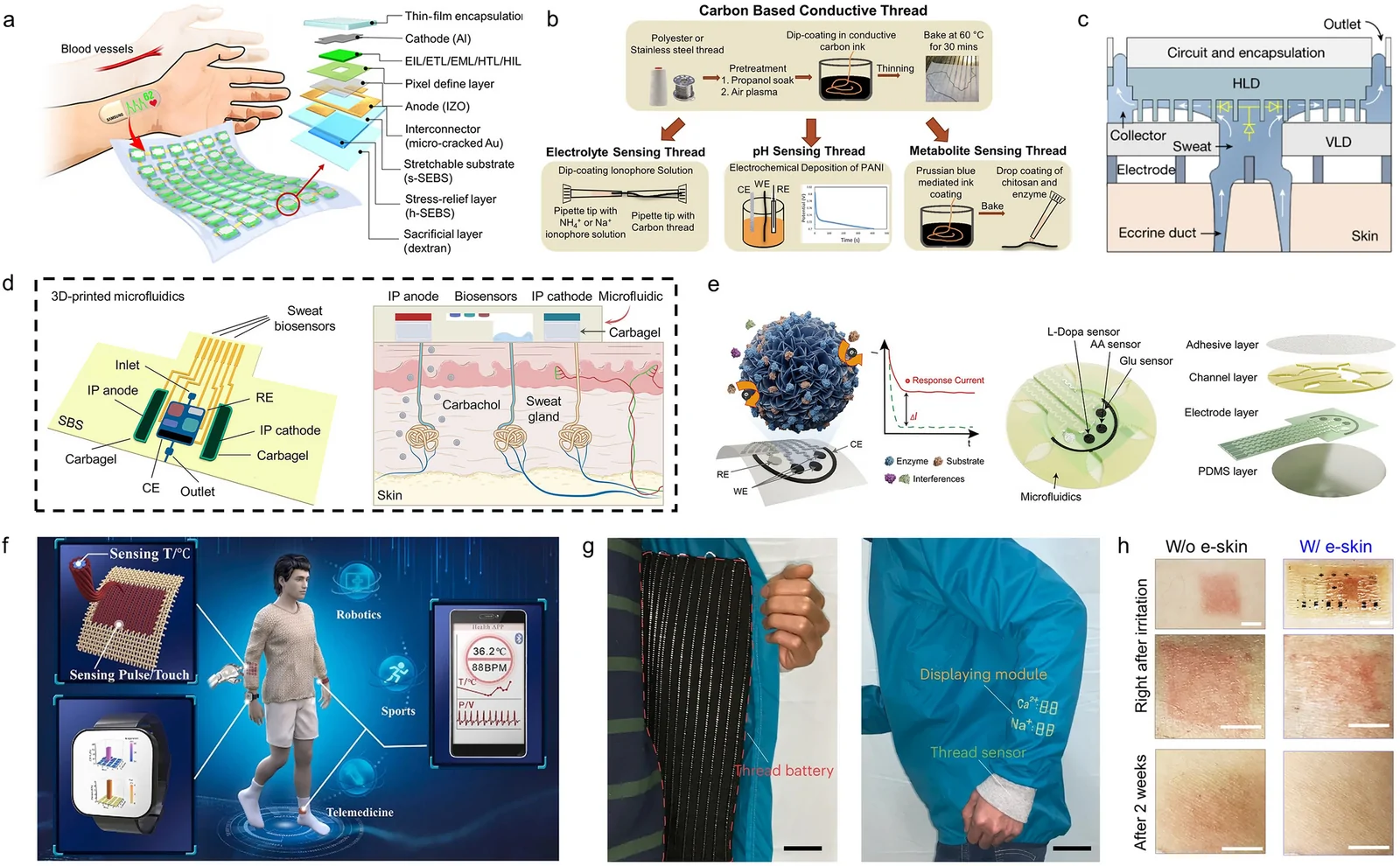
**a** Schematic of the skin-like health care patch attached to the forearm to measure the heart rate. Copyright 2021, The American Association for the Advancement of Science [47]; **b** Preparation of carbon-based conductive threads and sensing threads [49];** c** Illustration of the three-dimensional liquid diode. Reproduced with permission. Copyright 2024, Springer Nature [50]; **d** Design of the 3D-printed microfluidics for sweat induction, sampling, and multiplexed analysis [51]; **e** Schematic illustration of the integrated wearable sweat sensing patch. Reproduced with permission. Copyright 2025, John Wiley and Sons [53]; **f** Schematic illustration of the smart textile worn on the human body to simultaneously monitor temperature and pulse/touch with discriminability. Reproduced with permission. Copyright 2022, Elsevier [54]; **g** Photographs of the garment with the integrated textile system [58]. Scale bar, 10 cm. Reproduced with permission. Copyright 2024, Springer Nature;** h** Skin regeneration monitoring over a period of 2 weeks. “W/o e-skin” refers to naked skin without electronic skins, while “W/ e-skin” refers to skin covered by electronic skins. Scale bar, 5 mm. Copyright 2021, The American Association for the Advancement of Science [62]

Noninvasive flexible electronic devices, typically worn on the skin surface, represent the least intrusive class of health-monitoring technologies. Based on their working modality, these systems can be broadly divided into two categories [44, 45]. The first relies on direct physical contact with the epidermis [46]. Leveraging the intrinsic softness, stretchability, and conformability of polymeric substrates, such devices achieve intimate coverage of complex skin surfaces, thereby enabling continuous and high-fidelity sensing without breaching the skin barrier. For example, a 15-μm-thick skin-like health patch integrating a stretchable organic light-emitting diode display with a stretchable organic photoplethysmography sensor enables real-time heart-rate measurement and display [47] (Fig. 3a). Through robust skin–sensor coupling, these devices can capture both biophysical and biochemical signals [48]. Biophysical parameters are temperature, motion, pressure, strain, and electrophysiological activity (e.g., ECG, EMG, EEG). Meanwhile, biochemical signals can be acquired through sweat analysis, covering metabolites (e.g., glucose, lactate, cortisol), electrolytes, and pH [49] (Fig. 3b). To meet the critical requirement of breathability for practical applications, a three-dimensional (3D) liquid diode strategy was developed, offering superior long-term stability and user comfort even under sweating conditions, thereby underscoring its potential for scalable and user-friendly wearable devices [50] (Fig. 3c). Compared with conventional fabrics, the hydrophilicity-gradient channel rapidly pumps out sweat as droplets and accelerates their detachment from the back side. Increasingly, multimodal sensing systems that integrate both categories are being developed to provide comprehensive physiological profiling. Such noninvasive platforms show strong promise not only in fitness tracking, sleep quality assessment, hydration monitoring, and stress evaluation, but also in chronic disease management and telemedicine applications, where at-home continuous monitoring supports early diagnosis and remote clinical decision-making. For example, an individual’s behavioral responses can be accurately predicted through simultaneous monitoring of pulse waveform, temperature, and alcohol levels using machine learning-integrated electronic skins (e-skins) composed of multidimensional nanomaterials, polymers, and hydrogels. Such low-cytotoxicity 3D-printed biochemical sensors conformally adhere to the skin and exhibit excellent selectivity and stable performance under mechanical deformation [51] (Fig. 3d). By integrating multimodal flexible sensor patches with accelerometers, edge computing enables real-time detection of abnormalities such as arrhythmias, coughs, and falls [52]. Leveraging real-time detection of three key Parkinson’s disease biomarkers (levodopa, glucose, and ascorbic acid), a polyethylene terephthalate (PET)-based patch integrating Cu-oxidase hybrid nanoflowers can continuously monitor biomarkers, evaluate disease progression, and optimize medication management [53] (Fig. 3e).

The second category involves indirect or non-conformal modalities, in which signal acquisition does not depend on adhesive skin contact but rather on integration into textiles, accessories, or soft exoskeletal components. A smart textile system composed of thermally sensitive and conductive graphene/Fe_2_(MoO_4_)_3_/TPU fibers and nylon monofilament enables simultaneous mapping of temperature and pressure distributions at the contact interface [54] (Fig. 3f). Flexible photonic and thermal sensors embedded in garments or accessories can capture reflected or emitted signals without requiring close skin adhesion [55]. A dual-mechanism flexible iontronic pressure sensor composed of polyurethane (PU) and iontronic fabric delivers a linear capacitance–pressure response, enabling early fracture-risk prediction with only 1.8% error in ground-reaction-force estimation (vs. 6.5% for nonlinear sensors) [56]. Similarly, soft wearable sleeves or gloves integrating a thread battery, thread sensor, and thread electroluminescent device provide a mechanically coupled alternative to adhesive bonding for real-time biochemical monitoring and display. To address interfacial stability arising from highly curved textile surfaces in particle use, twisted fibers were employed, leveraging their intrinsic tensile tension to enhance contact robustness [57, 58] (Fig. 3g). These approaches offer enhanced wearability, unobtrusiveness, scalability, and long-term durability, making them particularly attractive for continuous monitoring in daily life. However, the lack of direct coupling introduces challenges such as motion-induced artifacts, baseline drift, and reduced sensitivity [59, 60]. Addressing these limitations requires material-level optimization (e.g., low-noise conductive polymers, breathable substrates) and system-level strategies [61].

In both categories, polymeric materials are central to device performance. Mechanically, they provide tunable elasticity and conformability for secure interfacing with skin [63, 64]. Functionally, they serve as active sensing components, including conductive polymers for electrophysiology, ion-sensitive hydrogels for biochemical monitoring, and nanocomposite films for signal enhancement [65–67]. Comfort and wearability are further improved through porous substrates and hydrogel-based contact layers, which reduce irritation during prolonged use [68]. In addition, emerging efforts focus on biodegradable and recyclable polymers, supporting both user safety and environmental sustainability [69]. Bioinspired adhesives, hydrogel-based interfaces, and microstructured elastomeric films are increasingly employed to enhance adhesion without sacrificing comfort [70, 71].

Although noninvasive devices are generally considered safe, long-term continuous wear may induce unintended effects, including irritation, sweat accumulation, and microbial growth—particularly under occlusive patches. Occlusion leads to a rapid increase in the local relative humidity (RH) at the skin–device interface, typically reaching 80%–95% within hours, increased transepidermal water loss (TEWL) fluctuation, and sweat accumulation [72]. These moisture-driven changes soften the skin, increase frictional irritation, and destabilize electrode–skin impedance, thereby reducing signal stability during long-term wear [73]. Microbiologically, Gram-negative bacteria typically require RH > 90%–95%, conditions rarely encountered on uncovered skin but readily achieved under occlusion. In contrast, Staphylococcus spp.—the dominant commensals in occluded environments—readily proliferate at RH as low as 81%–87% and develop enlarged cell size and thickened cell walls under low-water-activity conditions [74]. These shifts increase the likelihood of irritation and microbially driven discomfort. Therefore, vapor-permeable substrates, porous micro-vented designs, and antimicrobial or biofouling-resistant coatings are essential to maintain a breathable interface that limits RH excursions, preserves user comfort, and stabilizes epidermal impedance and improves noninvasive signal fidelity.

Beyond the development of new substrates—such as tunable polymeric materials including PET, polyethylene naphthalate (PEN), polyimide (PI), and elastomeric thermoplastic polyurethane (TPU)—even commercial products with high moisture vapor transmission rates and well-matched elasticity do not necessarily achieve improved user perception or long-term durability [75]. Addressing these risks has motivated the development of moisture-permeable substrates, antimicrobial coatings, and controlled release of skin-soothing agents, along with disposable or reusable device architectures. As an example, a sweat-pore-inspired perforated e-skin enables reliable 2-week monitoring without disturbing the skin’s recovery process [62] (Fig. 3h). While such strategies significantly improve comfort and safety, it remains challenging to ensure signal fidelity over extended timeframes, particularly in high-motion or high-sweat environments. More importantly, signal distortion or drift during long-term use may induce adverse psychological effects or contribute to the onset and aggravation of mental disorders. Although machine learning strategies can mitigate these issues to some extent [76–80], potential psychological implications remain underexplored in current literature, highlighting the need for interdisciplinary evaluation involving materials science, physiology, and mental health [81]. This bottleneck underscores the inherent trade-off of noninvasive modalities: While biologically safe, their sensing sensitivity and selectivity remain constrained by the barrier function of skin. Consequently, noninvasive devices, despite their progress, face fundamental bottlenecks in detecting low-abundance biomarkers and achieving precise molecular readouts. These limitations have driven growing interest in microinvasive modalities, which extend sensing depth while still aiming to minimize discomfort and biological risk. Importantly, the boundary between noninvasive and microinvasive systems is not discrete; instead, they exhibit a partial overlap in characteristic operational depth due to inter-individual and anatomical variations in epidermal thickness [82], the distributed nature of device penetration [83], and the ability of both modalities to probe near-surface physiological signals [84].

### Microinvasive Modality

**Fig. 4:**
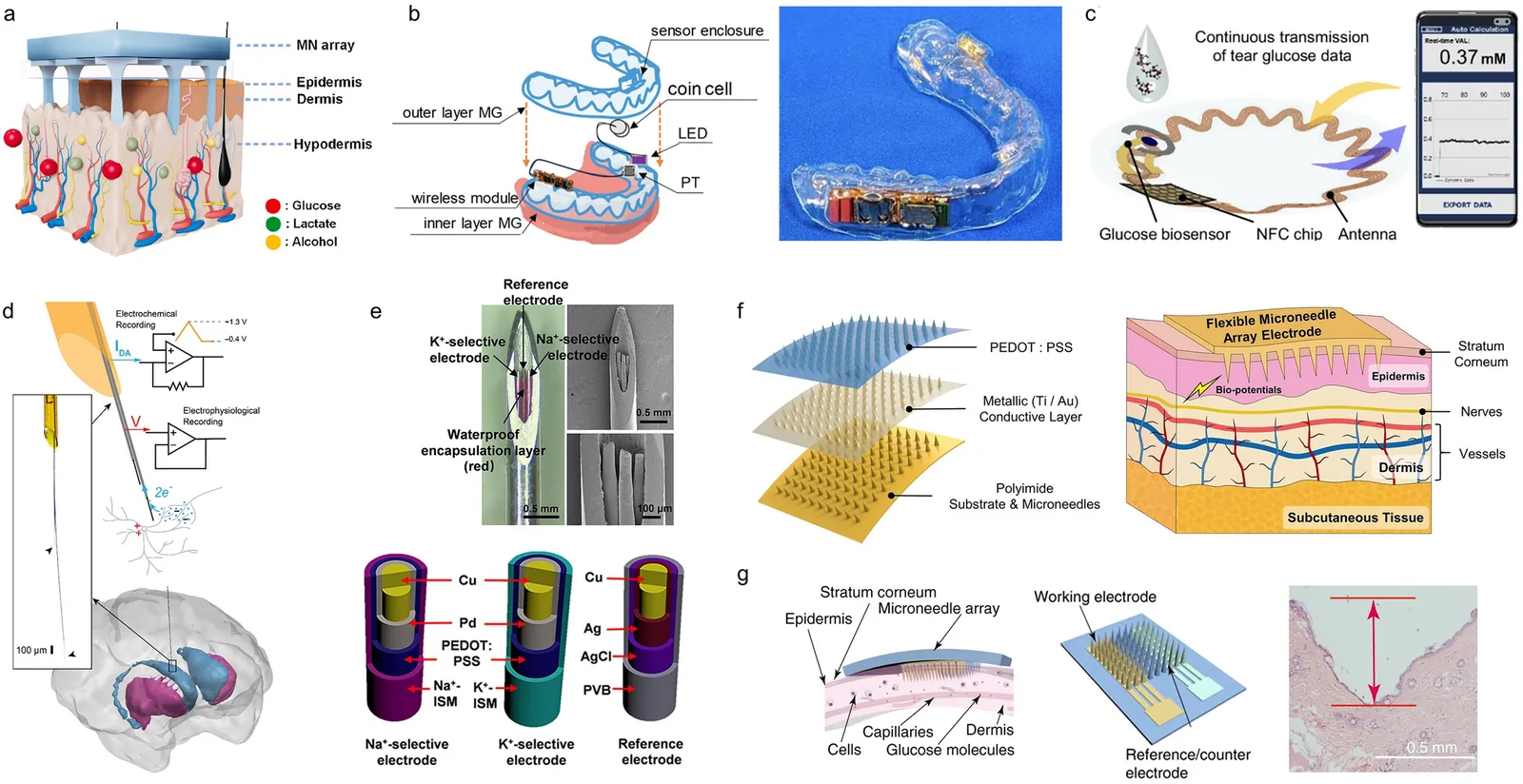
**a** A cross-sectional view of the microneedles piercing the skin to reach the epidermis. The penetration depth is approximately 1 mm. Reproduced with permission. Copyright 2024, Elsevier [108]; **b** Schematics and photograph of mouthguard-type salivary turbidity sensor [91]; **c** Schematic illustration of the tear glucose monitoring system using the smart contact lens and smart phone [93]; **d** Overview of the shaft-assisted microinvasive probes [95]; **e** An integrated potentiometric sensing system. Reproduced with permission. Copyright 2021, American Chemical Society [96]; **f** Microinvasive biopotential acquisition by a flexible MNA electrode. Reproduced with permission. Copyright 2024, Springer Nature [104]; **g** Illustration of the microneedle array inserted into the dermis and the optical image of the pierced skin [105]

Microinvasive flexible electronic devices represent an intermediate class of health-monitoring systems, situated between surface-worn wearables and fully implantable platforms (Fig. 4a). These devices partially penetrate biological barriers such as the stratum corneum, oral mucosa, or superficial subcutaneous tissues to access richer and more immediate biological signals, while minimizing pain, bleeding, and long-term tissue disruption [85]. Compared with noninvasive modalities, microinvasive systems provide enhanced biochemical sensitivity, particularly for interstitial fluid (ISF) biomarkers such as glucose, lactate, and cytokines. Their closer interfacing enables faster analyte transport, reduced signal delay, and improved temporal resolution, thereby enhancing both sensitivity and sampling stability [86, 87].

Representative microinvasive modalities can be broadly divided into three categories. Mucosa-interfacing patches are designed to adhere to oral, buccal, or nasal mucosa [88]. Utilizing bioadhesive hydrogel matrices or mucoadhesive polymers (e.g., contact lens, silicone elastomer), these patches form stable interfaces with moist tissues, enabling monitoring of saliva-based analytes such as electrolytes, cortisol, and inflammatory proteins [89, 90]. The wearable mouthguard-type sensor, encapsulated with polyethylene terephthalate-glycol (PETG) and ethylene–vinyl acetate (EVA), enables continuous and unobtrusive measurement of salivary turbidity, showing strong potential for real-time oral hygiene monitoring (Fig. 4b) [91]. This strategy also enables monitoring of salivary uric acid, a biomarker associated with various diseases. Specifically, an enzyme (uricase)-modified electrode system is integrated onto a flexible PET substrate via well-established screen-printing technology, resulting in a comfortable wearable device [92]. Flexible devices operating on the ocular surface mucosa can quantitatively monitor glucose levels in basal tears while excluding the influence of reflex tears, which may otherwise weaken the correlation with blood glucose (Fig. 4c). In this case, the lag time of glucose levels, captured by the electrochemical glucose biosensor, enables personalized assessment of an individual’s health status, benefiting from sub-minute resolution [93]. Soft bioelectronic threads or filaments, inserted subdermally through minimally invasive procedures, enable monitoring of temperature, electrophysiological activity, pH, or metabolites [94]. Their thread-like geometry allows conformal placement along dynamic tissues while ensuring ease of removal after short-term monitoring. Microinvasive probes have been developed to monitor electrical and chemical neural activity, consisting of metal rods and carbon fibers encapsulated with flexible parylene coatings to provide electrical insulation [95]. However, for monitoring electrical and chemical neural activity, it should be noted that to meet the requirements of primate recording chambers and the tube insertion protocols that must pass through the stiff outer meningeal membranes, the flexible portion had to be restricted to less than 15 μm. The remaining length was enclosed in a silicon tube to provide sufficient stiffness for manual handling (Fig. 4d). Conductive polymer coatings and polymer-based selective membranes have also been utilized to develop a microinvasive microneedle-based potentiometric sensing system. Such a system, which consists of a stainless-steel hollow microneedle housing a set of modified microneedle electrodes, exhibits rapid response, excellent reversibility and repeatability, and high selectivity for Na^+^ and K^+^ analysis in skin ISFs [96] (Fig. 4e). Microneedle arrays (MNAs) represent the most mature and widely adopted microinvasive approach [97, 98]. Constructed from swellable, degradable, or conductive polymers (e.g., polyvinyl alcohol (PVA), polyvinylpyrrolidone (PVP), poly(lactic-co-glycolic acid) (PLGA), poly(3,4-ethylenedioxythiophene)/polystyrene sulfonate (PEDOT:PSS) composites), microneedles enable passive ISF extraction, in situ biochemical sensing, and closed-loop integration with therapeutic delivery [99–103]. By avoiding contact with nerve endings and blood vessels, microneedles achieve minimal discomfort and high user compliance. For example, PI-based MNA (PI-MNA) electrodes exhibit high electrical (the electrode–skin contact impedance is roughly 1/250 that of standard electrodes) and mechanical performance while remaining compatible with wearable wireless recording systems [104]. The combination of comfort and stability enables clinical long-term continuous monitoring for polysomnography (Fig. 4f). For prevalent chronic metabolic diseases such as diabetes, MNA biosensing devices enable continuous glucose monitoring with performance comparable to commercial blood glucose meters. With a penetration depth limited to only 0.5 mm, these devices offer a minimally invasive alternative that may open new avenues for diabetes monitoring and management [105] (Fig. 4g).

Within these modalities, polymeric materials underpin critical design trade-offs. Mechanically, tips must be stiff enough to penetrate skin yet compliant enough to avoid irritation. Composite strategies such as core–shell structures and shape-memory polymers are commonly used to balance these requirements [106]. Biocompatibility is addressed through degradable polymers such as PLGA, gelatin methacrylate, and silk fibroin, which enable benign degradation or safe removal [107]. Functionally, conductive polymers facilitate electrophysiological recording and electrochemical readouts, while hydrophilic networks enhance ISF absorption and ionic transport. Increasingly, anti-biofouling or hemocompatible coatings are applied to mitigate protein adsorption and infection risk, while stimuli-responsive polymers provide on-demand insertion, dissolution, or signal transduction.

Compared with noninvasive devices that often suffer from motion-induced artifacts and delayed analyte transport, microinvasive modalities inherently provide improved temporal fidelity and reduced baseline drift, albeit at the cost of introducing new biocompatibility challenges and unique safety concerns. Repeated applications may cause localized erythema or inflammation, and mucosal deployment carries elevated infection risk. Moreover, incomplete dissolution or material retention presents additional hazards if device degradation is not tightly controlled. Strategies to address these challenges include antimicrobial coatings, swellable insertion–retrieval mechanisms, and fully bioresorbable architectures [109]. However, achieving consistent safety across diverse users and environments remains challenging, and current regulatory frameworks for microinvasive systems are less established compared with fully implantable devices [110].

Overall, microinvasive polymer-based devices offer a valuable compromise between safety and signal richness, providing direct access to biomarkers that remain inaccessible to surface wearables. They show particular promise for real-time glucose monitoring, point-of-care diagnostics, and temporally resolved stress biomarker tracking. Yet, their long-term reliability and standardization remain open challenges. Future progress will likely emphasize closed-loop therapeutic integration, wireless communication modules, and adaptive polymeric platforms capable of dynamically responding to physiological environments, alongside the development of regulatory guidelines to accelerate clinical translation.

### Short-term Implantable Modality

Short-term implantable devices are designed for acute and time-limited clinical needs, operating within hours to several weeks and targeting postoperative states, transient dysfunction, and early-phase physiological changes. Functionally, their purpose is to provide high-fidelity access to local biological environments during short therapeutic windows, after which the device is safely removed or fully bioresorbed.

Short-term implants are typically deployed in subcutaneous, intra-organ, or mucosal sites for postoperative monitoring, acute disease detection, or temporally localized therapeutic feedback [111]. Representative applications can be broadly grouped into four categories: (a) monitoring wound healing or inflammation after surgery [112]; (b) acquiring short-term electrophysiological signals in neural or cardiac tissue; (c) mapping pressure, temperature, pH or other biomarkers within localized internal regions of the body; and (d) assessing drug response in localized cancer therapy [113]. Each category corresponds to a distinct short-term clinical need and leverages polymeric materials in different ways.

**Fig. 5:**
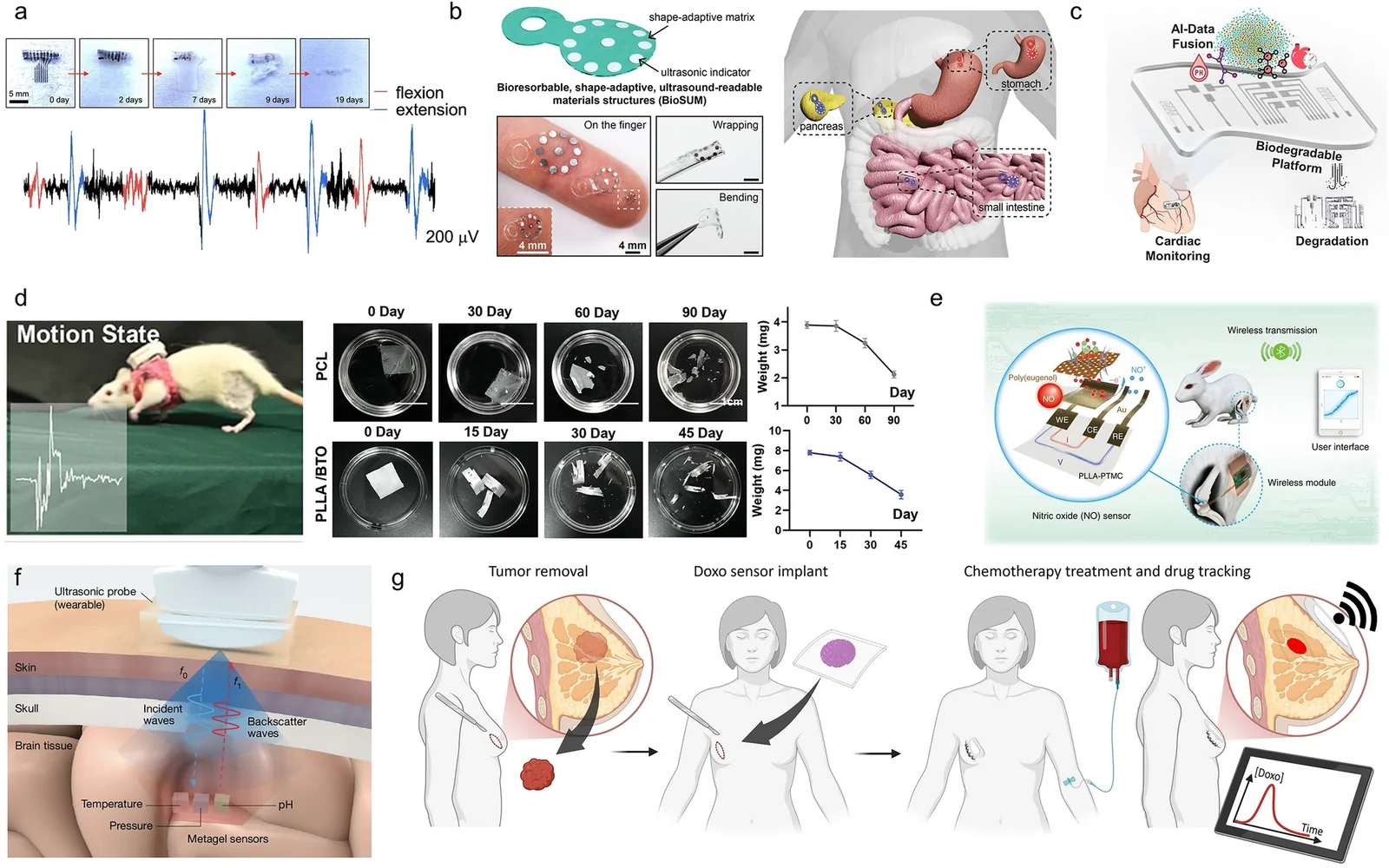
**a** Biodegradable, restorative, and self-morphing neural interface. Accelerated dissolution was performed in PBS at pH 7.4 and 60 °C [114]; **b** Bioresorbable shape-adaptive ultrasound-readable material structures for real-time monitoring of homeostasis in deep tissues. Reproduced with permission. Copyright 2025, Springer Nature [115];** c** Biodegradable multiplex nanosensor platform for cardiac monitoring [116]; **d** Evaluation of the motion function in vivo and the photographs of the degradable condition of PCL films and PLLA/BTO. Reproduced with permission. Copyright 2024, John Wiley and Sons [117];** e** Materials and designs for flexible and transient NO sensors [118]; **f** Diagram showing metagels as wireless intracranial physiology sensors using ultrasound reflection. Reproduced with permission. Copyright 2024, Springer Nature [119]; **g** Concept of in vivo monitoring of doxorubicin in tissue using a bioresorbable sensor coupled with a wearable readout patch. Reproduced with permission. Copyright 2025, The American Association for the Advancement of Science [120]

In the first category, a biodegradable and restorative neural interface has been designed to enable concurrent monitoring and facilitation of long-gap nerve repair within a defined timeframe aligned with the critical initial phase of nerve recovery (Fig. 5a). The use of flexible, biodegradable polymer substrates and shape-memory polymer films (poly(l-lactic acid) (PLLA) and poly(trimethylene carbonate) (PTMC)) facilitates surgical placement of the device onto injured nerves [114]. Beyond relying on costly imaging modalities or invasive biopsies, arrays of small bioresorbable metal disks embedded within hydrogels enable real-time monitoring of deep-tissue homeostasis using conventional ultrasound instruments [115]. Collections of small bioresorbable metal disks embedded in thin, pH-responsive hydrogels enable ultrasound-based monitoring of spatiotemporal pH changes for early detection of anastomotic leaks after gastrointestinal surgery. Importantly, although the hydrogel–metal constructs may require up to several weeks for complete bioresorption, the operational sensing window remains confined to the early postoperative period, eliminating the need for surgical removal (Fig. 5b).

In the second category, short-term electrophysiological monitoring is achieved using biodegradable sensor arrays. For instance, a biodegradable sensor array fabricated from PLLA (approximately 1-year degradation time in simulated body fluid, SBF) with biodegradable magnesium (degradation within 24 h in SBF) and nanoparticles (two-month degradation in SBF), enabling detection of multiple cardiac-related biomarkers, including pressure, lactic acid, pH, and volatile organic compounds [116] (Fig. 5c). Here, the active monitoring duration remains within days to weeks, despite the slower degradation of certain structural components. To address the challenge of real-time evaluation of motor function recovery following nerve injury, an implantable piezoelectric sensor fabricated from degradable PLLA has been developed (Fig. 5d). By doping with barium titanate (BTO), the platform converts biochemical signals into electrical outputs that correlate with EMG signals and motor function [117].

In the third category, short-term implants map local biochemical and physicochemical environments. Precisely capturing nitric oxide (NO)—an essential mediator in neurotransmission, immune responses, cardiovascular regulation—in physiological environments is highly challenging due to its short half-life, low concentration, high chemical reactivity, and interference from other biomolecules (e.g., glucose, nitrites, and uric acid) [118]. To address this, a flexible and degradable platform has been developed, offering a low detection limit (3.97 nmol), high temporal resolution, and wide sensing range (0.01–100 μM) over a period exceeding 5 days. Equipped with a wireless module, the device enables real-time monitoring of NO evolution in cultured cells and organs, providing critical information for health assessment, treatment optimization, and postsurgical monitoring (Fig. 5e). A bioresorbable and wireless metastructured hydrogel (metagel) sensor for ultrasonic monitoring of intracranial signals has been fabricated using biodegradable, stimulus-responsive hydrogels combined with periodically aligned air columns [119]. This metagel sensor enables independent detection of intracranial pressure, temperature, pH, and flow rate and achieves a detection depth of up to 10 cm, while the construct undergoes complete degradation after ~ 18 weeks. Importantly, the sensing function is restricted to the acute-to-subacute postoperative period (Fig. 5f).

In the fourth category, short-term implants assess local drug response in cancer therapy. Such systems provide a route to balancing therapeutic efficacy and toxicity through precise, real-time drug monitoring at the target site, thereby enabling clinicians to optimize dosing during cancer treatment and reduce the risk of locoregional recurrence after tumor resection [120]. Take the pharmacokinetics of doxorubicin (chemotherapy medication used to treat cancer) as an example, the biodegradable sensor based on bioreceptor and PLGA foil achieves a limit of detection of 3 ng mL^−1^ and no signs of systemic toxicity, histological, or biochemical alterations over a 3-month period. Despite the long evaluation timeframe, the device’s active sensing window remains confined to the early therapeutic phase (Fig. 5g).

Together, these representative applications highlight the versatility of short-term implantable devices across structural, electrophysiological, biochemical, and pharmacological domains. Across these diverse use cases, devices must remain biocompatible and non-inflammatory during the operational window, while permitting safe removal or complete bioresorption after use. Relative to microinvasive strategies, short-term implants offer higher signal fidelity due to their stable embedding in tissue and direct access to less-diluted biofluids such as blood, lymph, or organ-specific exudates. Additionally, mechanical isolation from surface motion artifacts improves stability of signal acquisition [121].

Polymers used in short-term implants must offer a finely tuned balance between mechanical robustness and temporary biostability. The defining requirement is that their functional integrity matches the clinically relevant window of hours to several weeks, irrespective of the total degradation duration. Bioresorbable polymers, such as PLLA, PLGA, polycaprolactone (PCL), and citrate-based elastomers, are particularly attractive due to their ability to degrade within hours to days or even weeks depending on molecular weight, crystallinity, and pH [122–124]. For temporary electrophysiological monitoring, transient conductive materials are embedded in degradable matrices or silk–metal hybrids. Hydrogel-based sensors also play a prominent role in vivo, where ionic or enzymatic sensing elements can be embedded within cross-linked hydrogel scaffolds for detecting metabolites, cytokines, or drug concentrations. Meanwhile, thin polymer encapsulation layers such as polyimide or polyurethane enable temporary isolation of wireless modules, which can be retrieved or dissolved following operation.

Degradation kinetics critically shape the short-term safety of transient implants. Bulk-eroding polymers such as PLGA and citrate-based elastomers (e.g., poly(octamethylene maleate (anhydride) citrate), POC) absorb water throughout the matrix and undergo autocatalytic hydrolysis, producing localized acidic microenvironments that accelerate modulus loss and may trigger transient inflammation [125, 126]. In contrast, surface-eroding systems such as poly(glycerol sebacate) (PGS) and polyanhydride (PAH) degrade in a layer-by-layer manner, offering linear and geometry-preserving mass loss with minimal pH drift [127, 128]. These differences highlight the need to report mass loss curves, modulus retention, and local pH trajectories under physiologically relevant conditions and to employ buffering fillers, hydrophilic comonomers, or porosity control to moderate erosion-induced safety risks.

Although transient implants circumvent many chronic risks inherent to permanent systems, the acute responses arising within hours to days demand equally rigorous material safety considerations [129]. Local immune responses triggered by device insertion, toxic degradation by-products, or early device failure due to unstable barrier layers remain important concerns. Tissue encapsulation or adhesion during the monitoring period can also complicate device removal. These risks highlight the importance of designing polymers with predictable degradation kinetics, non-toxic metabolites, and reliable mechanical integrity within the intended operational window [130, 131].

Future directions for short-term implants will likely emphasize fully biodegradable wireless systems, eliminating the need for surgical retrieval. Self-powered devices using enzymatic biofuel cells or thermoelectric harvesters may further enhance practicality. Modular device architectures that allow controlled breakdown, on-demand retrieval, and multimodal sensing–therapy integration are also promising. However, a key challenge remains in balancing device robustness with safe degradation: Excessive stability risks tissue encapsulation, while premature breakdown compromises data reliability. Addressing this trade-off will be crucial for clinical adoption, and bridging this gap will require close alignment of degradation kinetics with clinically relevant monitoring windows, as well as standardized in vivo models for preclinical validation.

### Long-term Implantable Modality

By contrast, long-term implantable devices in this review refer to platforms designed for chronic and progressive medical conditions, requiring continuous and stable operation for periods exceeding approximately three months. Their purpose is to support sustained physiological monitoring or closed-loop therapy in dynamic biochemical and mechanical in vivo environments, where durable biostability, immune modulation, and long-term mechanical and electrochemical reliability are essential.

Long-term implantable devices target fundamentally different clinical objectives from short-term systems, enabling persistent monitoring in chronic disease management [132], neural and cardiovascular monitoring [133], and closed-loop therapeutic systems [134]. Unlike transient or microinvasive platforms, these devices must withstand prolonged exposure to chemically aggressive biofluids, cyclic mechanical loading, and evolving immune activity, while maintaining stable signal coupling and functional integrity—often without the possibility of device retrieval. Representative platforms include chronic brain–computer interfaces, long-term glucose or ion-homeostasis monitors, soft tissue strain sensors for ligament reconstruction, and implantable vascular sensors capable of multi-month hemodynamic assessment. Collectively, these applications require reliable performance over multi-month to multi-year timescales, well beyond the acute therapeutic windows addressed by short-term implants.

**Fig. 6:**
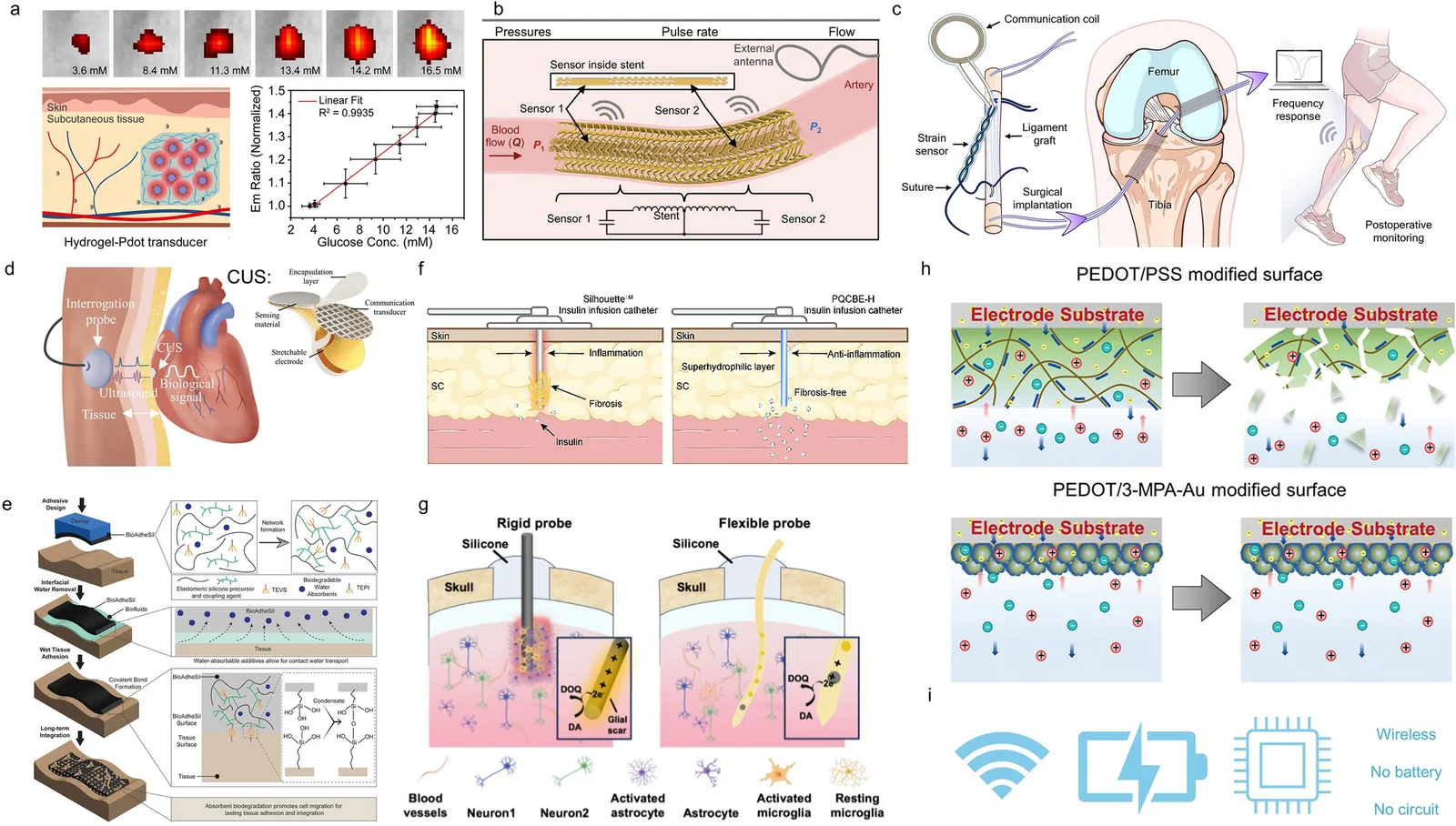
**a** Implantable hydrogel platform embedded with luminescent polymer dots for sensitive and long-term glucose monitoring. Reproduced with permission. Copyright 2022, American Chemical Society [135]; **b** Illustration of the wireless design and sensing scheme to simultaneously monitor pressure, heart rate, and flow. Reproduced with permission. Copyright 2022, The American Association for the Advancement of Science [136]; **c** Implantable wireless sensor for tendon and ligament strain monitoring through in situ suturing. Reproduced with permission. Copyright 2025, The American Association for the Advancement of Science [137]; **d** Schematic of monitoring physiological pressure signals inside the human body via the circuit-free ultrasonic system (CUS) [145];** e** Cross-linking mechanism and adhesive chemistry of silicone-based bioadhesive for bonding silicone devices to biological tissues. TEVS, triethoxy vinyl silane; TEPI, triethoxy(3-isocyanatopropyl)silane [159]; **f** Fibrosis-resistant implants allow infusion catheters to be used long term and enable faster insulin absorption. Reproduced with permission. Copyright 2024, Elsevier [148]; **g** Schematic diagram of flexible and rigid probe implanted in brain tissue. Reproduced with permission. Copyright 2024, American Chemical Society [151]; **h** Illustration of conductive coatings during the stability test. Reproduced with permission. Copyright 2024, John Wiley and Sons [156]; **i** Design principles of long-term implantable devices for health monitoring

To illustrate, an implantable hydrogel platform embedded with luminescent polymer dots has been developed for sensitive, long-term glucose monitoring, exhibiting no migration and maintaining intrinsic sensing properties with excellent stability for up to one month [135]. The extraordinary brightness of the polymer dot transducers enables real-time in vivo glucose monitoring through transdermal optical signal detection (Fig. 6a). For vascular diseases, a hemodynamic monitoring system has been constructed based on a wireless stent platform integrated with PI, silver nanoparticles, and polydimethylsiloxane (PDMS) to form stretchable, soft pressure sensors (Fig. 6b). In an arterial model, the wireless system monitors changes in systemic pressure, flow rate, and pulse rate, as well as sudden abnormal fluctuations [136]. To address the monitoring of postoperative complications from severe sports injuries, a strain sensor with a double-helix configuration—based on PEDOT:PSS and encapsulated with poly(chloro-para-xylylene) (parylene-C)—has been developed, demonstrating accurate monitoring capability and stable performance within the 0–10% strain range with a minimum detection threshold of 0.25% [137]. As illustrated in Fig. 6c, the wireless sensor can be integrated into complex surgical constructs for lateral collateral ligament repair or anterior cruciate ligament reconstruction, enabling distinct responses to graft stretching, reinjury, and loosening. Despite their distinct clinical contexts, all three systems exemplify the demand for durable, stable sensing across extended recovery periods and long-term disease management windows.

However, long-term use inevitably introduces complications such as chronic immune response, fibrotic encapsulation, biofouling, signal drift, and material fatigue. At the micro- and nanoscale, long-term biocompatibility is largely determined by the earliest interfacial events. Zwitterionic hydrogels suppress nonspecific protein adsorption and macrophage recruitment, enabling fibrosis-free implantation in vivo [138]. PEG–phosphorylcholine hydrogels further show that soft, tissue-like moduli yield significantly thinner capsules than stiffer networks [139]. Surface micro/nanotopography also regulates macrophage behavior: engineered patterns that induce elongated morphology bias cells toward anti-inflammatory M2 phenotypes and reduce collagen deposition and FBR severity [140–142]. Mechanically robust zwitterionic hydrogels integrate these chemical and structural cues, mitigating chronic inflammation and maintaining long-term interface stability [143]. In addition, compliant ultrathin polymer substrates help preserve tissue–device mechanical coupling and suppress strain-transfer instability, a major precursor of chronic impedance drift and long-term signal degradation in dynamic organs [40]. Together, these findings highlight that surface chemistry, wettability, and topography at the first tens to hundreds of nanometers critically determine capsule thickness, impedance drift, and long-term signal fidelity.

Polymeric materials provide a wide toolbox to address these challenges. Encapsulation polymers such as parylene-C offer excellent barrier properties, preventing fluid ingress while maintaining flexibility [144]. PI provides robust thermal and mechanical stability, and medical-grade silicone elastomers are valued for elasticity and oxygen permeability, though they are less effective barriers against small molecules [145]. The encapsulation of the stretchable electrode layer, piezoelectric transducer, and sensor within a PDMS layer enables reconstruction of physiological signals by analyzing the characteristics of reflected ultrasound pulses. Because ultrasound exhibits much lower attenuation in the human body (− 1 dB cm^−1^) compared with electromagnetic waves (− 3 dB cm^−1^), the system supports a communication depth of up to 14 mm in living organisms (Fig. 6d). While inert encapsulation remains a dominant strategy, there is a growing recognition that complete biological isolation is insufficient [146]. Instead, controlled tissue integration—for example, porous elastomers enabling partial cellular infiltration—has been shown to reduce fibrotic encapsulation and improve long-term signal coupling. As shown in Fig. 6e, a silicone-based bioadhesive has been formulated to provide robust adhesion between hydrophobic silicone devices and hydrophilic tissues by mixing soft silicone oligomers with siloxane coupling agents and absorbents. The incorporation of biodegradable and water-absorbent additives (like starch or carbopol) removes surface water and regulates porosity, while silane cross-linkers enhance interfacial strength. Over time, the bioadhesive transitions from non-permeable to permeable through enzyme-mediated degradation, generating a porous structure that promotes cell migration and tissue integration, thereby enabling durable adhesion. Another strategy is exemplified by zwitterionic polymers, such as poly(sulfobetaine methacrylate) (PSBMA), which can resist protein fouling and macrophage adhesion by forming strong hydration shells, significantly enhancing interface stability [147]. With in situ-generated pure zwitterionic surfaces, a coating-free elastomer that is hydrophobic in bulk exhibits superior performance as a fibrosis-resistant implant for up to six months of use (Fig. 6f). Specifically, this superior performance is achieved by hindering the initial stage of the immune cascade through resistance to non-specific protein adsorption [148]. Together, these encapsulation and interface-engineering strategies address the chronic challenges unique to long-term implantation, including fibrosis, persistent immune activation, and long-term signal drift.

Mechanical mismatch remains another critical issue. Soft tissues such as brain or myocardium exhibit bending stiffness orders of magnitude lower than most electronic materials [149]. To address this, ultrathin polymer films (e.g., PI, parylene-C, PDMS) below 10 µm in thickness can achieve bending stiffness comparable to biological tissue, reducing shear stress at the interface [150]. An ultraflexible electrode modified with a nanocomposite of reduced graphene oxide and PEDOT:PSS establishes a stable electronic interface, enabling simultaneous detection of neural electrical activity and dopamine concentrations deep within the brain [151] (Fig. 6g). Flexible polymers including hydrogels, shape-memory polymers, and liquid crystal elastomers accommodate cyclic organ motion without delamination or cracking [152–154]. However, despite advances, achieving true long-term mechanical compliance remains an unsolved problem, particularly for highly dynamic environments like the heart or bladder. Long-term mechanical adaptability therefore remains one of the central bottlenecks to multi-year implantable systems.

For reliable signal acquisition, conductive elements must remain electronically and chemically stable for years under physiological conditions. PEDOT and PEDOT:PSS are attractive for their softness and low impedance, but they still suffer from gradual dedoping and loss of conductivity in the physiological environment [155]. Density functional theory calculations indicate a robust Coulomb interaction between PEDOT and 3-mercaptopropionic acid (3-MPA), surpassing PEDOT:PSS. As reported, a hybrid film composed of 3-MPA-modified gold nanoparticles and PEDOT exhibits superior electrochemical and mechanical stability, enabling the capture of high-quality, long-term electrophysiological signals in vivo and continuous recording of target neurons for up to 16 weeks [156] (Fig. 6h). Carbon-based materials such as graphene and carbon nanotube (CNT)–polymer composites show promise for enhanced durability, though challenges in reproducible large-scale fabrication persist [157]. Hybrid metal–polymer systems, combining ultrathin noble metals with stretchable substrates, have shown success but still face risks of fatigue and corrosion [158]. These advances collectively highlight the necessity of developing conductive systems capable of maintaining low-impedance, high-stability operation over months to years.

Despite these advances, long-term implants remain the most demanding modality, requiring simultaneous solutions to mechanical, biochemical, and immunological challenges [160]. Device failure modes—including delamination, fibrotic encapsulation, biofouling, and material degradation—are not fully eliminated by current polymer strategies [161]. Furthermore, regulatory approval for long-term implantable polymers remains stringent, demanding rigorous demonstration of stability, safety, and reproducibility. Moreover, the field’s understanding of long-term in vivo performance remains incomplete, and careful consideration is required to balance the benefits and risks of lifelong or multi-year implantation. For healthy individuals, the necessity of long-term implants for health monitoring is questionable. By contrast, an application-oriented design principle is far more critical for guiding the development of long-term modalities [162]. Looking forward, the design of long-term implantable systems will increasingly rely on multifunctional polymer interfaces that integrate robust encapsulation with immune modulation, anti-biofouling performance, and mechanical adaptability—capabilities essential for operation beyond three months and into multi-year timeframes. Wireless operation with battery-free or circuit-free designs is essential for developing long-term in vivo platforms with minimal risk (Fig. 6i). The integration of AI-driven monitoring and adaptive therapeutic feedback may also become a key direction, enabling devices that not only record signals but actively respond to the body’s evolving state [163]. Importantly, the molecular tunability and structural versatility of polymers provide a unique opportunity to engineer such interfaces across chemical, mechanical, and biological dimensions. Yet, translating these laboratory advances into clinically validated systems that operate reliably over years and across human-scale variability remains the ultimate bottleneck. Ultimately, the success of long-term implantable systems will depend not only on advanced polymer chemistry but also on their ability to demonstrate reliable, reproducible, and clinically validated performance over multi-month to multi-year operation.

## Safety-level-oriented Framework Integrating Functional Layers and Polymeric Material Classes

**Fig. 7:**
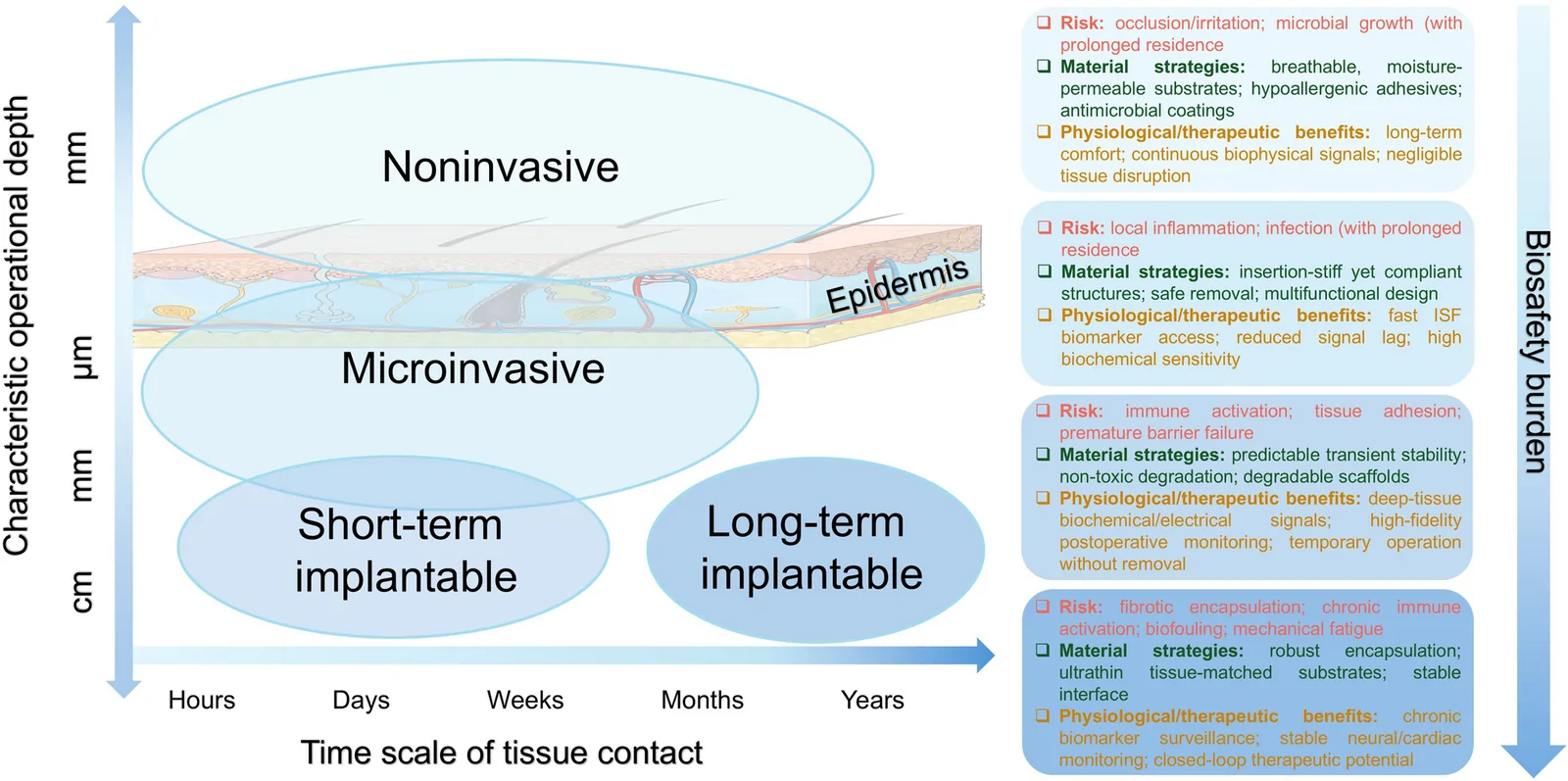
Safety-level-oriented mapping of polymer-based health-monitoring modalities across operational depth, tissue contact time, and biosafety burden

A safety-level-oriented framework enables polymer-based health-monitoring devices to be systematically positioned along a continuum from noninvasive to long-term implantable systems (Fig. 7). This continuum reflects not only the physical depth of device deployment but also the escalating biological burdens imposed by tissue contact time, biomechanical coupling, and immunological engagement. Such a perspective is essential for flexible polymer-based electronics, whose soft mechanics, biointegration pathways, and degradation behaviors extend well beyond the categorizations traditionally used for rigid medical devices. By structuring the field around operational depth and physiological residence time, the framework provides a mechanistic basis for comparing device classes that would otherwise be difficult to evaluate within a single conceptual space.

At the noninvasive level, devices remain external and impose minimal biological risk. Their interface function relies on soft, breathable, and biocompatible elastomers or hydrogels to ensure comfort and stable skin coupling [164]. Longer tissue contact time introduces risks of occlusion, irritation, and microbial growth, which necessitate the use of porous or moisture-permeable films, antimicrobial coatings, and hypoallergenic adhesives [165]. In microinvasive modalities, which penetrate only superficial tissues or mucosa, both interface and acquisition functions require mechanical precision and mild biocompatibility. As tissue contact time increases, the likelihood of local inflammation and infection also rises. This concern motivates the use of bioresorbable microneedles, zwitterionic hydrogels, and antimicrobial coatings to support reliable signal capture while minimizing immune response. At greater tissue depths and residence times, the biological burden increases sharply, necessitating the shift into short-term implantable modalities. Short-term implantable devices, embedded for hours to several weeks, face elevated risks of immune activation, tissue adhesion, and premature failure of protective layers. These modalities require materials that can sustain acquisition and feedback functions during transient but direct contact with internal tissues. Polymers in this category must exhibit predictable short-term stability and non-toxic degradation, with bioresorbable scaffolds and drug-eluting hydrogels frequently employed. Long-term implantable systems, designed for continuous operation over weeks to years, encounter the most stringent interface, acquisition, and feedback requirements. Persistent tissue contact time increases the risks of fibrotic encapsulation, chronic immune activation, biofouling, and mechanical fatigue. To address these challenges, long-term implantable devices rely on robust encapsulation materials, ultrathin tissue-matched substrates, electrochemically stable conductive polymers, and zwitterionic coatings to maintain both functionality and biocompatibility.

**Fig. 8:**
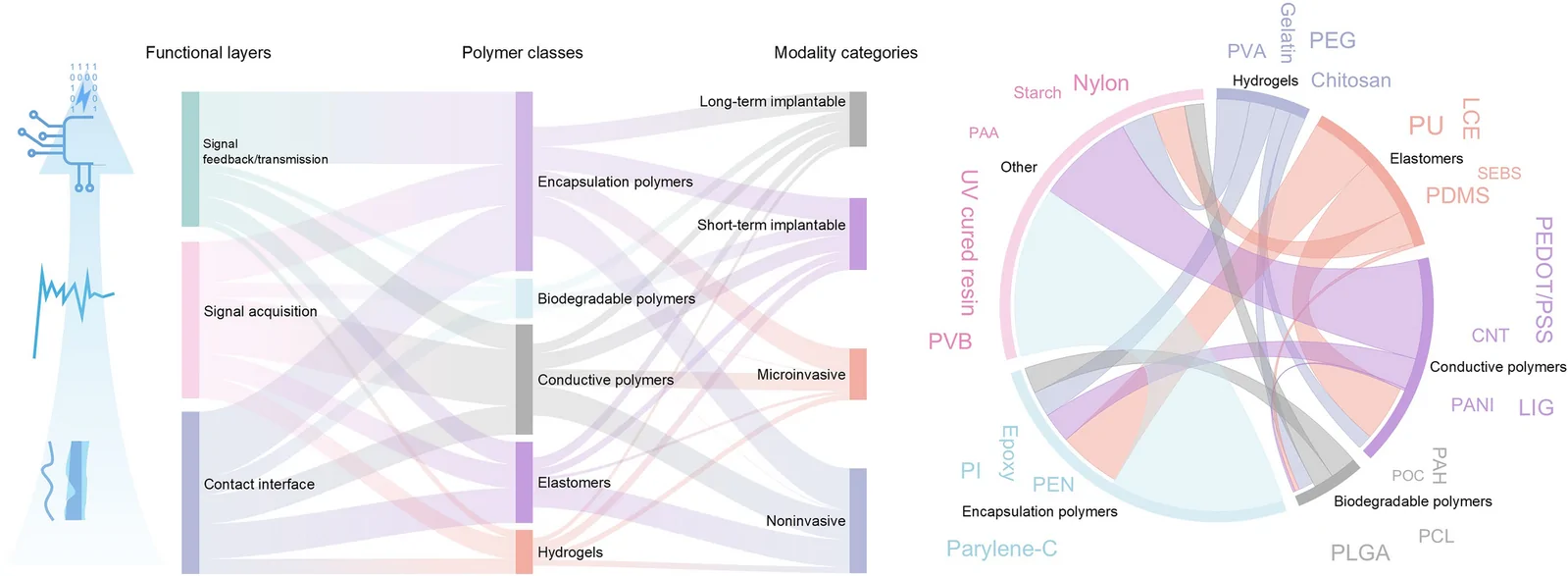
Integrated mapping of functional layers, polymer material classes, and modality categories in flexible health-monitoring systems. PEG: poly(ethylene glycol); SEBS: styrene–ethylene–butylene–styrene block copolymer; LCE: liquid crystal elastomer; CNT: carbon nanotube; PANI: polyaniline; LIG: laser-induced graphene; PAA: poly(acrylic acid); PVB: poly(vinyl butyral)

While this classification is fundamentally governed by invasiveness and tissue contact time, the functional requirements of each modality, including contact interface, signal acquisition, and signal feedback/transmission, directly shape the selection of polymeric material classes incorporated within these devices. These functional layers and material classes together provide a mechanistic basis for understanding how biosafety constraints influence polymer design. To further contextualize how polymeric materials operate with these constraints, Fig. 8 illustrates the relationships among functional layers, polymer material classes, and the modality categories. This mapping highlights the sequential nature of material selection in polymeric health-monitoring systems. Each modality draws on a characteristic combination of interface, acquisition and feedback functions, and these functional demands guide the use of specific polymer families. Hydrogels and elastomers predominantly support interface functions in noninvasive and microinvasive systems due to their softness and tissue-matched mechanics, whereas conductive polymers and encapsulation materials become increasingly important for acquisition and feedback operations as devices move toward short- and long-term implantation. Several polymer systems also demonstrate multifunctionality within this landscape. Conductive hydrogels, ionic elastomers, and other hybrid systems contribute simultaneously to more than one functional layer, enabling them to operate across adjacent safety levels and facilitating smoother transitions between sensing regimes [166, 167].

Taken together, these observations complement the safety-level-oriented framework by clarifying how polymer classes align with the operational workflow of health-monitoring devices. This integrated perspective illustrates that although tissue contact time modulates material demand, the degree of invasiveness remains the primary determinant of biosafety. The progression from hours to years imposes escalating demands on stability, immune compatibility, and long-term functional persistence. This reinforces that tissue contact time and invasiveness jointly dictate biosafety thresholds and shape the feasible material space for each modality. While ISO 10993-1 provides internationally recognized categories for medical-device biocompatibility, its structure—organized around contact type and duration—does not capture the multilayered interplay among device architecture, polymer chemistry, functional roles, and operational depth that defines flexible polymer-based electronics. Rather than replacing ISO standards, our safety-level-oriented framework complements them by offering a modality-specific, materials-integrated perspective uniquely suited to emerging soft, transient, and multifunctional devices, for which traditional classifications provide insufficient resolution. In this sense, the framework functions not only as a taxonomic tool but also as a forward-looking design map for emerging soft and multifunctional polymeric systems.

By positioning diverse modalities within this unified structure, we provide guiding principles for rational polymer design and device architecture that support a balanced combination of safety, durability, and functional performance. This framework also clarifies critical challenges that remain unresolved, including the need for standardized metrics for long-term biocompatibility and the persistent difficulty of reconciling biodegradability with stable signal transduction. Addressing these gaps will be essential for advancing next-generation polymeric systems capable of supporting reliable, long-term physiological monitoring.

## Perspectives and Outlooks

Looking ahead, the development of polymer-based health-monitoring modalities will be guided by several critical directions.

1. Strengthening an application-oriented design philosophy will be essential. Polymers must be selected not only for their intrinsic properties but also for their compatibility with specific usage scenarios. Hydrogels, for example, offer excellent biocompatibilities and ionic conductivity but are hindered by dehydration and mechanical fragility; in contrast, solvent independent elastomers provide more robust alternatives for long-term external monitoring. On the other hand, in vivo applications, dehydration can be reasonably neglected due to the aqueous physiological milieu. Under these conditions, hydrogel-based systems gain distinct advantages due to their tissue-like water content, tunable porosity, and biochemical functionalization, enabling superior biointegration and signal fidelity. This contrast illustrates the importance of aligning material choice with the specific operating environment rather than adhering to a one-size-fits-all paradigm.
2. Careful transition from conventional metals to flexible semiconductors and polymer circuits represents both an opportunity and a challenge. Emerging organic semiconductors, stretchable conductors, and polymeric processors promise seamless integration of sensing, processing, and communication within soft devices. However, their reliability and maturity still lag behind established metal-based technologies. In the near term, selective hybridization-combining polymeric components with metallic counterparts may provide the most pragmatic pathway toward clinical transition, balancing innovation with proven stability.
3. Advancing biodegradable and transient systems will open new opportunities for short-term implantable and microinvasive platforms. Bioresorbable polymers such as PLGA, PCL, and silk fibroin enable devices that naturally degrade after use, eliminating the need for surgical retrieval and reducing long-term risks. Coupling such materials with transient electronics and resorbable power modules could transform temporary monitoring after surgery or acute disease into a safe, fully self-resolving intervention.
4. System-level integration of power supply, sensing, and data communication is required in a unified platform. While current flexible health-monitoring devices often rely on bulky external batteries or wired connections, future systems are expected to adopt on-body power solutions such as thin-film stretchable batteries, energy harvesting modules (thermoelectric, triboelectric, or piezoelectric generators), and wireless power transfer. Meanwhile, sensing modalities are moving from single-parameter acquisition to multimodal platforms capable of simultaneously recording electrophysiological, mechanical, and biochemical signals. To fully realize their potential, these sensing units must be seamlessly coupled with wireless communication modules, such as flexible antennas and near-field communication circuits. Such strategies enable continuous and secure transmission of physiological data. The convergence of power, sensing, and transmission into polymer-based flexible systems will mark a crucial step toward practical, autonomous, and clinically relevant health-monitoring technologies.
5. Personalization and scalable manufacturing are expected to play a decisive role in bridging laboratory research and real-world adoption. Additive manufacturing, electrospinning, and textile integration allow the customization of device geometry, porosity, and mechanics to match individual patient need; at the same time, scalable production strategies are necessary to ensure reproducibility, regulatory compliance, and cost-effectiveness.
6. Establishing standardized safety metrics within the biosafety framework will be critical. Although polymers provide diverse options for tailoring mechanical compliance, degradation behavior, and interfacial chemistry, systematic evaluation of their biosafety across modalities remains lacking. Future research must prioritize quantitative benchmarks for immune response (e.g., macrophage activation, fibrosis indices), standardized degradation windows aligned with clinical monitoring timelines, and longitudinal signal stability metrics. Harmonizing these parameters into a common framework will not only enable more rigorous comparison of different material platforms but also accelerate regulatory approval and clinical translation. By integrating safety metrics directly into material design and device evaluation, the safety-level-oriented paradigm can evolve from a conceptual framework into a practical roadmap for polymer selection and device certification.
7. Ensuring long-term stability remains a central bottleneck across all four modalities. Despite differing levels of invasiveness, polymer-based systems ultimately fail through similar mechanisms, including interfacial drift, fibrotic encapsulation, moisture-induced degradation, mechanical fatigue, and progressive impedance or signal decay. Future progress will benefit from a streamlined design–testing pathway that includes: i material-level evaluation of modulus matching, permeability, and degradation kinetics; (ii) interface-level assessment of adhesion, strain-transfer stability, and fouling resistance; (iii) device-level accelerated aging under physiologically relevant humidity, ionic strength, and cyclic loading; and (iv) staged in vivo validation aligned with the four safety levels. Embedding such a systematic pathway within the proposed safety-level-oriented framework will be essential for achieving reliable long-term physiological monitoring.

Taken together, these perspectives emphasize that the future of polymer-based health monitoring will not be defined by a single material breakthrough, but by the rational integration of application-driven material selection, judicious adoption of new electronic paradigms, safe transient designs, and personalized manufacturing strategies. Within this safety-level-oriented framework, polymers are uniquely positioned to enable monitoring systems that are not only flexible and biocompatible but also adaptive, durable, and clinically translatable—ultimately fulfilling the promise of a safety-level-oriented framework for polymer-based health monitoring.
